# People who use drugs engagement in substance use disorder services and harm reduction: evaluation, challenges and future direction of a community-based intervention

**DOI:** 10.1186/s13011-024-00601-1

**Published:** 2024-04-30

**Authors:** Julie Gleason-Comstock, Cindy Bolden Calhoun, Barbara J. Locke, Naga Vijaya Lakshmi Divya Boorle, Kevin Cobty, Tiffany McKenney, Kaji O. Uddin, Samantha J. Bauer, Jinping Xu

**Affiliations:** 1 Department of Family Medicine & Public Health Sciences, School of Medicine, Wayne State University, Detroit, MI USA; 2 Community Health Awareness Group, Inc, Detroit, MI USA; 3 Department of Family Medicine & Public Health Sciences, Public Health Research Lab (PHRL), Wayne State University, Detroit, MI USA; 4 Wayne State University School of Medicine, Detroit, MI USA

**Keywords:** People who use drugs, Substance use disorder, Harm reduction, Community-based organization, Evaluation, Social determinants of Health

## Abstract

**Background:**

Since 1996, an urban community-based organization whose primary mission is to serve diverse94 and emerging community health needs has provided screening, testing, overdose prevention and training, referrals, and access to treatment for substance use disorders (SUD) and communicable diseases such as HIV through its Life Points harm reduction program.

**Methods:**

As a partner in a State survey in 2021, the community organization recruited a convenience sample of people who use drugs to participate in a survey focused on their substance use, healthcare, and barriers to SUD services. Community health workers conducted outreach and used an encrypted identifier to collect data from a convenience sample of harm reduction participants regarding demographics, legal justice, engagement in harm reduction and access to healthcare. Evaluators entered paper surveys into Qualtrics for reporting and summative analysis.

**Results:**

A convenience sample of fifty-five people who use drugs were recruited and surveyed. The majority (86%, *n* = 47) were active participants in the agency Life Points (LP) harm reduction service. Participants’ average age was 42.9 years (SD = 11.5). About half (51%, *n* = 28) were male, 48% (*n* = 26) were female, and 2% (*n* = 1) was transgender. About two-thirds (67%, *n* = 37) of participants were White/Caucasian, 13% (*n* = 7) were Black/African-American, 11% (*n* = 6) were Hispanic and 7% (*n* = 4) were Multi-Racial. Regarding current substance use, 98% (*n* = 54) reported use of heroin, 51% (*n* = 28) reported crack, 47% (*n* = 26) cocaine, 25% (*n* = 14) alcohol, 24% (*n* = 13) opioids, and 15% (*n* = 8) marijuana. The majority, 87% (*n* = 48) said they had health care insurance and over two-thirds (69%, *n* = 37) said they had been arrested for a felony. Almost three quarters (71%, *n* = 39) reported receiving services from the Department of Health & Human Services. A higher percentage of females compared to males (65% and 29% respectively) reported engagement in community mental health services and 69% of females (*n* = 18) compared to 15% (*n* = 4) of males reported needing to participate in sex to meet basic social needs. Participants described social determinants of health as barriers to services, including access to food, legal justice and transportation. About 44% (*n* = 24) said they would consider enrolling in a drug treatment program in the next 30 days.

**Conclusion:**

This sample was reflective of increased participation by White participants that began to appear about a decade ago. The majority of participants reported having healthcare insurance, which may be reflective of engagement with community health workers to access appropriate services. Community organizations and healthcare professionals should continue to explore social determinants of health that can impact the health of people who use drugs, including overcoming barriers to health care access such as investing in mobile unit outreach.

## Background

Literature examining disparities related to SUD highlight the factors that influence treatment utilization and outcomes across various demographic groups, including race, age, and gender. Drug toxicity mortality rates in the United States (US) among racial and ethnic groups (except for non-Hispanic Asian), different biological sexes, and age groups of 25 years and older have all increased in recent years [1]. Research found that individuals experiencing substance use disorders (SUD) are often associated with a lower quality of life compared to the general population or those with other chronic health conditions [2].

Perceived treatment needs are lower for minority racial and ethnic groups, such as African-Americans and Latinos, who tend to underestimate their personal treatment needs compared to White/Caucasian individuals [3]. This difference is correlated to disparities in SUD treatments and overall health. Limited access to SUD treatment is influenced by environmental factors. Studies have found that the effective integration of SUD treatment into primary care and hospital settings is limited due to a lack of SUD-related training for healthcare providers [4]. Rural and urban areas also present significant disadvantages to underserved communities, including a lack of essential services and underutilization of available resources. These challenges are emphasized by limited facilities and public transportation options, impacting the clients’ access to care [5–7].

Among people with drug use, stigma is also a challenge for accessing health-related services, including non-prescription syringes. Stigma operates at both individual and systemic levels. For example, enacted stigma from experiences of discrimination and dismissive attitude and behavior from health care providers, including pharmacists, may leave left a negative perception affecting engagement and utilization of these services [8, 9]. However, people with drug use reported a more comfortable experience during their interaction with safe syringe programs when those experiences are provided by community health workers who may have a more non-judgmental approach and positive attitude [10]. Harm reduction strategies aimed at mitigating the adverse consequences of drug use have gained increasing attention from public health stakeholders. A potential benefit of this attention is that harm reduction strategies can overcome other known barriers to combating drug overdose, such as individuals’ fears of engaging with healthcare and emergency systems to systemic issues in healthcare and emergency medical response systems [11].

Incorporating harm reduction initiatives within inpatient facilities offers a unique opportunity to connect inpatient and outpatient care, thereby expanding harm reduction services [12]. However, challenges related to stigma, power dynamics, and role coordination among care team members were identified as barriers to successful implementation with mental health disorders and untreated SUD often experience an increased risk of chronic physical conditions due to mental illness, untreated SUD, and physical health outcomes [13]. Access to basic needs such as stable housing, nutritious food, and employment opportunities becomes difficult, exacerbating the health disparities they already encounter. Additionally, navigating the criminal justice system poses a significant challenge for many in these communities, which can lead to a cycle of incarceration rather than addressing the underlying issues of mental health and substance use.

These disparities highlight the need for increased support for harm reductionists to reach those communities. Advocating for systemic changes, such as increased funding for treatment of SUD as a part of holistic healthcare can contribute to systems that promote the long-term health of people struggling with substance use [10]. In addition to in-patient facilities, the use of mobile healthcare is a potential resource to expand SUD treatment. Mobile health clinics have been designed to provide pension screening and triage [14], increase patient accessibility [15] and provide follow-up services [6]. They have proven to be effective for health screening and education [6, 7]. Making services available through outreach including mobile health units may enhance substance use disorder services to persons who use drugs.

The community-based organization has previously collaborated with other community organizations and health departments to identify communities disproportionately affected by HIV and populations at high risk, who may not have access to traditional testing services [16, 17]. Utilizing mobile testing units and vehicles equipped with private counseling spaces was an effective way to bring HIV counseling and rapid testing directly to at-risk populations, such as street corners, homeless shelters, and community special events, where the most participants were tested. This method ensured that HIV testing reached individuals who may otherwise go untested, which ultimately contributed to HIV prevention efforts. Logs were collected to record clients’ demographics and harm reduction services received, including condoms for males and females, syringes, wound and hygiene kits, and referrals. Analyzing data from community-based harm reduction and service referrals was conducted to determine social determinants of health [18–22].

## Methods

This SUDS study describes a convenience sample of people who use drugs who self-reported access to healthcare, participation in human services and engagement in harm reduction. Life Points protocol and evaluation research in risk reduction provided the foundation for the SUDS study. Community health workers did community outreach, collected data on Life Points Logs and conducted the survey. The Life Points protocol provided an encrypted identifier with coded de-identified data. Additional Life Points Log variables included race, sex, risk behavior, supplies (i.e., condoms, hygiene kits). Multiple Life Points Harm Reduction Outreach Project survey questions of residence, employment, education, drug treatment preference, healthcare status, life satisfaction, criminal justice status and barriers to treatment were incorporated into the survey.

The final questions were reviewed in collaboration with a State Working Group. The SUDS survey protocol was reviewed and determined by the Wayne State University (WSU) Institutional Review Board to in the category of Program Evaluation/Quality Improvement/Quality Assurance (#2021-096) and Non-Human Participation Research.

Community health workers used the encrypted Life Points identifier for participants and completed paper surveys. Survey data of demographics, legal justice parameters, engagement in harm reduction and access to healthcare was gathered. The anonymous survey data was provided to the evaluators for secondary data analysis. WSU evaluators entered paper surveys into Qualtrics as a secure WSU database for summative analysis using SPSS 27.

## Results

Fifty-five people who use drugs were recruited and interviewed by seven community health workers working from Community Health Awareness Group (CHAG) central office and mobile health units, where 60% of the interviews (*n* = 33) took place. The average age of participants was 42.9 years (SD = 11.5) in a range of 28–73 years old: 51% (*n* = 28) were male, 48% (*n* = 26) female and 2% (*n* = 1) transgender. 67% (*n* = 37) were White/Caucasian, 13% (*n* = 7) were Black/African-American, 11% were Hispanic (*n* = 6), and 7% (*n* = 4) were Multiracial. The majority were not employed (73%, *n* = 40) and most reported single marital status (67%, *n* = 37). Please see Table 1. Demographics.

**Table 1:** Demographics

	Gender			Total
Characteristic (*)	Male *n* = 28	Female *n* = 26	Transgender *n* = 1	*N* = 55
Race/ethnicity (**)				
White/Caucasian (non-Hispanic)	15 (54%)	21 (81%)	1 (100%)	37 (67%)
Black/African-American (non- Hispanic)	6 (21%)	1 (4%)	--	7 (13%)
Multiracial**	3 (11%)	1 (4%)	--	4 (7%)
Hispanic*** (of all races)	4 (14%)	2 (8%)	--	6 (11%)
Age mean (SD), range	45.9 (SD = 13.09) 28–73 years	40.2 (SD = 8.85) 29–57 years	31	42.9 (SD = 11.52) 28–73 years
Highest Grade Completed				
9th grade	1 (4%)	2 (8%)	--	3 (6%)
10th grade	4 (14%)	1 (4%)	--	5 (9%)
11th grade	2 (7%)	6 (23%)	--	8 (15%)
12th grade	12 (43%)	12 (46%)	--	24 (44%)
Trade School	1 (4%)	--	--	1 (2%)
Some college	6 (21%)	5 (19%)	1 (100%)	12 (22%)
College graduate	1 (4%)	--	--	1 (2%)
GED	14 (50%)	9 (35%)	--	23 (42%)
Employment				
Full time	6 (21%)	2 (8%)	--	8 (15%)
Part time	2 (7%)	2 (8%)	--	4 (7%)
Not working	17 (61%)	22 (85%)	1 (100%)	40 (73%)
Retired	2 (7%)	--	--	2 (4%)
Veteran Status				
Yes	4 (14%)	--	--	4 (7%)
No	24 (86%)	25 (96%)	1 (100%)	50 (91%)
Marital Status				
Single	17 (61%)	19 (73%)	1 (100%)	37 (67%)
Married	4 (14%)	2 (8%)	--	6 (11%)
Separated	--	2 (8%)	--	2 (4%)
Widowed	1 (4%)	1 (4%)	--	2 (4%)
Divorced	5 (18%)	1 (4%)	--	6 (11%)
Committed Couple	1 (4%)	1 (4%)	--	2 (4%)

* Excludes missing data and some participants provided multiple responses to a question

Column percentages calculated based on gender-specific totals

** 4 individuals self-reported multiple race/ethnicities. Multiracial included Black/African-American and Hispanic (n = 1), White/Caucasian and Hispanic (n = 1), White/Caucasian and Other (n = 2)

***Per the NIH and CDC, individuals could identify by race and also the Hispanic ethnicity

The majority (87%, *n* = 48) of these individuals who self-reported as people who use drugs also reported having healthcare insurance. Additionally, when asked if they had a personal doctor or healthcare provider, 50% (*n* = 27) said yes. Nonetheless, 47% (*n* = 26) of these individuals also visited the emergency room: 15% (*n* = 8) said once in the last year and 33% (*n* = 18) said 2–5 times in the last year. Please see Table 2. Access to Healthcare.

**Table 2:** Access to Healthcare

	Gender			Total
Characteristic	Male *n* = 28	Female *n* = 26	Transgender *n* = 1	*N* = 55
Health care Insurance				
Yes	25 (89%)	22 (85%)	1 (100%)	48 (87%)
No	1 (4%)	3 (12%)	--	4 (7%)
Don’t know/Not sure	1 (4%)	--	--	1 (2%)
Healthcare Provider				
Yes	14 (52%)	13 (50%)	--	27 (50%)
No	11 (41%)	12 (46%)	1 (100%)	24 (44%)
Don’t know/Not sure	1 (4%)	1 (4%)	--	2 (4%)
COVID-19 Testing				
Yes	19 (71%)	18 (69%)	--	37 (67%)
No	8 (29%)	8 (31%)	1 (100%)	17 (31%)
COVID-19 Vaccination				
Yes	11 (42%)	10 (39%)	--	21 (39%)
No	15 (58%)	16 (62%)	1 (100%)	32 (60%)
Emergency room visits in the last year				
None	16 (57%)	11 (42%)	1 (100%)	28 (51%)
Once	4 (14%)	4 (15%)	--	8 (15%)
2–5 times	7 (25%)	11 (42%)	--	18 (33%)

Column percentages calculated based on gender-specific totals.

Participants could choose multiple responses to self-report current substance use. Almost all (98%, *n* = 54) reported use of heroin. About half (51, *n* = 28%) reported crack and cocaine (47%, *n* = 26). Around a quarter reported use of alcohol (25%, *n* = 14) and opioids (24%, *n* = 13). Less than a fifth reported marijuana use (15%, *n* = 8). There could be a relationship between the responses to cocaine and crack in that crack cocaine is made when powder cocaine is processed into a rock form so that it can be smoked. Please see Table 3. Drug Use by Gender.

**Table 3:** Drug Use

	Gender			Total
Drug	Male *n* = 28	Female *n* = 26	Transgender *n* = 1	*N* = 55
Heroin	27 (50%)	26 (48%)	1 (2%)	54 (98%)
Crack	11 (39%)	17 (61%)	--	28 (51%)
Cocaine	12 (46%)	13 (50%)	1 (4%)	26 (47%)
Alcohol	8 (57%)	6 (43%)	--	14 (25%)
Opioid	5 (39%)	8 (62%)	--	13 (24%)
Marijuana	5 (63%)	3 (38%)	--	8 (15%)
Other*	6 (60%)	4 (40%)	--	10 (18%)

*Other: Weed and Zany Bars *n* = 4, Crank *n* = 2, Speed *n* = 2, Speedball *n* = 1, Meth *n* = 1

Note: Participants could provide multiple responses regarding substance use

Row percentages calculated based on substance-specific totals; total column percentages calculated based on participant total (*n* = 55)

Participants were asked if hunger or access to nutritious food, housing status, engagement in sex for basic needs or legal justice had been a barrier to substance use services. Hunger and access to food were reported by half of respondents (50%, *n* = 28), followed by legal justice (44%, *n* = 23), engagement in sex for basic needs (43%, *n* = 23) and housing status/unhoused (29%, *n* = 16). Please see Table 4. Barriers to Substance Use Disorder Services.

**Table 4:** Barriers to Substance Use Disorder Services*

	Gender			Total
	Male *n* = 28	Female *n* = 26	Transgender *n* = 1	*N* = 55
Experiencing hunger or lack of access to nutritious food				
Yes	12 (43%)	15 (58%)	1 (100%)	28 (51%)
No	15 (54%)	11 (42%)	--	26 (47%)
Legal Justice				
Yes	14 (52%)	8 (33%)	1 (100%)	23 (44%)
No	13 (48%)	16 (67%)	--	29 (56%)
Engagement in sex for money, housing, or other basic needs				
Yes	4 (15%)	18 (69%)	1 (100%)	23 (43%)
No	23 (85%)	8 (31%)	--	31 (57%)
Housing Status				
Yes	6 (21%)	10 (39%)	--	16 (29%)
No	20 (71%)	15 (58%)	1 (100%)	36 (66%)

*Excludes missing data

Column percentages calculated based on gender-specific totals

About three-quarters (73%, *n* = 40) reported receiving harm reduction services or services through the Department of Health & Human Services (71%). Less than half said they currently received treatment of SUD (45%), and about a third said they had participated in employment services such as Michigan Works (35%) and Community Mental Health (31%). Throughout all of those services, participation was about evenly divided between males and females, with the exception of community mental health, in which females reported more participation. Please see Table 5. Engagement in Human Services and Harm Reduction.

**Table 5:** Engagement in Harm Reduction and Human Services*

	Gender			Total
Characteristic	Male *n* = 28	Female *n* = 26	Transgender *n* = 1	*N* = 55
Harm Reduction/SSP				
Yes	20 (36%)	20 (77%)	--	40 (73%)
No	8 (53%)	6 (23%)	1 (7%)	15 (27%)
Department of Human Services/DHS				
Yes	17 (44%)	21 (54%)	1 (3%)	39 (71%)
No	11 (69%)	5 (31%)	--	16 (29%)
Substance Use Disorder Services				
Yes	10 (40%)	14 (56%)	1 (4%)	25 (45%)
No	18 (60%)	12 (40%)	--	30 (55%)
Michigan Works				
Yes	9 (47%)	9 (47%)	1 (5%)	19 (35%)
No	19 (53%)	17 (47%)	--	36 (66%)
Community Mental Health/CMH				
Yes	5 (29%)	11 (65%)	1 (6%)	17 (31%)
No	23 (61%)	15 (39%)	--	38 (69%)

*Respondents reported access to multiple services

Row percentages calculated based on engagement-specific totals; total column percentages calculated based on participant total (*n* = 55)

Participants were given additional opportunity to qualitatively describe barriers or factors that can create barriers to SUD services, including legal justice, food access, engagement in sex for basic needs, and housing status.

### Legal justice

- Over two-thirds, 69% (*n* = 37) of participants reported having been arrested for a felony.
- About a third of respondents, 34% (*n* = 16) reported having had interactions with legal authorities within the last year, another third, 34% (*n* = 16) reported having had interactions within the last 5 years. Slightly less than a third, 32% (*n* = 15) reported that they had not interacted with legal authorities for more than 5 years.
- More than half (56%, *n* = 29) reported that their interaction with legal authorities was not a barrier to engaging with substance use services.

### Food access

- 52% (*n* = 28) of participants reported experiencing hunger or lack of access to nutritious food.
- Participants expressed food access challenges and described hunger leading to drug use, substance-induced hunger, and a lack of motivation to obtain food.
- Panhandling, spending most of their money on drugs instead of food and relying on soup kitchens for meals were also mentioned.

### Engagement in sex

- 43% (*n* = 23) of participants reported engaging in sex work as a means to address basic needs such as food and housing.
- One respondent reported that engagement in sex for basic needs makes it difficult for her to achieve self-improvement because it prevents her from “getting herself together.”
- Participants reported engaging in sex to acquire drugs, which related to the cycle of lifestyle challenges such as financial instability and compromised mental health that often accompany chronic drug use due to its impact on a person’s physical, mental, and social well-being.

### Homelessness

- 31% (*n* = 16) of participants described experiencing homelessness (also described as unhoused) due to the persistent uncertainty surrounding their living situations.
- The lack of a stable, secure residence led to inadequate access to nutritious food and a safe environment.

### Other barriers (personal identification and transportation)

- 40% (*n* = 22) of respondents reported facing challenges related to identification cards. The absence of official identification impeded access to healthcare services and benefits and could also be a barrier to obtaining employment or housing.
- 36% (*n* = 20) of respondents reported transportation as a barrier to accessing healthcare services and employment, especially for those residing in areas with limited public transportation.

### Harm reduction frequency and activity

Participants were asked by the community-based organization about their harm reduction activity and frequency of service. They were asked if they could receive free supplies, whether those should be available weekly or monthly. About three-quarters (76%, *n* = 42) said they would prefer receiving free supplies weekly and 22% (*n* = 12) said they would prefer receiving them monthly.

Most said syringe exchange (95%, *n* = 52) was most important for harm reduction access, however a substantial percentage also said crack/meth pipes (71%, *n* = 39) and condoms (60%, *n* = 33). The agency distributes both male and female condoms. Female and transgender respondents were more likely to report the importance of condoms. Please see Table 6. Access to Harm Reduction.

**Table 6:** Access to Harm Reduction*

	Male *n* = 28	Female *n* = 26	Transgender *n* = 1	Total *N* = 55
Syringes	27 (96%)	25 (96%)	--	52 (95%)
Crack/Meth pipes	19 (68%)	19 (73%)	1 (100%)	39 (71%)
Condoms	11 (39%)	21 (81%)	1 (100%)	33 (60%)

*Respondents reported access to multiple services

Column percentages calculated based on gender-specific totals

As previously noted in Tables 6 and 60% of respondents noted condoms as a harm reduction activity, suggesting the need for further exploration around sexuality and people who use drugs. When asked if they were living with someone with whom they had a sexual relationship, about half, 53% (*n* = 29) said yes and over a third, 38% (*n* = 21) said their sexual partner also injected drugs. Please see Table 7. Harm Reduction and Sexual Partners.

**Table 7:** Harm reduction and sexual partners

	Male *n* = 28	Female *n* = 26	Transgender *n* = 1	Total *N* = 55
Are you currently living with someone with whom you have a sexual relationship?				
Yes	17 (61%)	11 (42%)	1 (100%)	29 (53%)
No	11 (39%)	15 (58%)	--	26 (47%)
Does your (sexual) partner also inject drugs?*				
Yes	12 (43%)	9 (35%)	--	21 (38%)
No	9 (32%)	10 (39%)	1 (100%)	20 (36%)
Don’t know	1 (4%)	--	--	1 (2%)

*13 individuals did not respond

Column percentages calculated based on gender-specific totals

## Discussion

Survey participants for this study were reflective of a small convenience sample of the community organization’s harm reduction program. Drug use, average age, and gender distribution was similar to a larger cohort of the community organization’s over 3,700 harm reduction participants registered between 2009 and 2013. The majority of that larger cohort of agency participants are Black/African-American, reflective of the urban city population in which the community organization is based. That sample also includes a greater representation of the LGBTQIA + community, which is important for examining the correlation between discrimination and heightened odds of developing substance use disorders among LGBTQIA + adults [23]. The majority of respondents to this survey were White/Caucasian and only had one LGBTQIA + participant, therefore limiting the demographic scope of the survey. The convenience sample was about half male, half female, with one person identifying as transgender. These three groups were demographically fairly similar across variables with the exception that more females reported engagement in sex for basic needs, and more males reported participation in legal justice. A higher percentage of females also said they were engaged in community mental health and syringe services programs which seek to reduce the harm associated with drug use and prevent HIV and viral hepatitis infections. Further study is warranted to determine how specific human services might be driven by gender considerations.

Research is underway to review data of people who use drugs and were registered in the agency harm reduction program between 2013 and 2023 to further analyze participant demographics and social determinants of health in key areas such as healthcare access, social and community context, and neighborhood and built environment. Specific examples of social determinants of health related to these areas are access to nutritious foods, safe housing and neighborhoods, and transportation. The community-based organization and its health workers work closely with the persons who use drugs to encourage them to enroll in health insurance, whether public insurance, such as Medicaid or the state-sponsored health insurance program, known as Healthy Michigan Plan, or other private health insurance. For a larger study, the access to health insurance would likely be dependent upon the health service relationships that persons who use drugs have chosen to pursue, or have chosen not to enroll in health insurance.

The agency is exploring the possibility of enhancing the billing capacity for community health workers who have completed their Michigan Community Health Worker Alliance (MiCHWA) certification. Although both the agency home base and CLIA waivered mobile units have the capacity to provide behavioral health services which include substance use disorders,, we there is a need to increase the billing capacity to support and sustain this initiative.

### Limitations

Strength of the study is demonstrated by the ability of the community health workers to conduct a survey with persons who use drugs. The study, however, has certain limitations which include being a convenience sample of a limited number of respondents interviewed over the period of a month. The convenience sample was recruited through an intervention which has a much larger database, for which analysis has not been completed due to limited funding over the period of a decade for evaluation research. Additionally, the limited funding resources and timeline for the survey did not permit a six-month follow-up with participants, which could have been helpful to determine factors such as whether they went into drug treatment.

### Conclusion

This convenience sample was reflective of increased participation by White participants that began to appear about a decade ago. Although the majority of people who use drugs reported having healthcare insurance, they didn’t seek medical treatment for drug use disorder but did participate in harm reduction activities. Community organizations, including community health workers and healthcare professionals, should continue to explore social determinants of health that can impact the health of people who use drugs, including investing in mobile unit outreach and outreach to young adults to provide risk reduction and primary prevention resources.

## Public health implications and future direction

Social determinants of health as identified by Healthy People 2030 identify healthcare and education access and quality, social and community context, economic stability and neighborhood and built environment as key areas [24]. As demonstrated in this study, the realities of people who use drugs intersects with all of these elements.

This analysis is part of a larger study that has collected data on harm reduction and drug use behaviors since 2000. Based on an evaluation registration database established with the academic partner, 2416 registrations and 3823 encounters from Life Points were documented between 2000 and 2012. Research data entry and analysis of registrations from 2013 to 2023 is underway and include this convenience sample of 55 participants.

Future Direction could also incl implications from SUD for young adults, the next generation of people who use drugs. The community organization conducts outreach and provides multiple services to young adults at risk, and has determined consideration should also be given to preparation of targeted services to youth and their families. This data is documented in the Life Points and other community health worker Daily Logs for which analysis is underway. A family history of substance use, adverse childhood events such as abuse or trauma, mental health disorders, low self-esteem, LGBTQIA + identity or same-sex relationships were all identified as factors that amplify the cultural or environmental risks associated with substance use and addiction in adolescents [25].

Our study supports other research that has shown substance use treatment and harm reduction are essential components for beneficial behavioral comprehensive lifestyle strategies for people who use drugs [10]. Effective January 2024, the Michigan Department of Health and Human Services announced an expansion of Medicaid coverage to include community health worker services. The community health workers will focus on the social determinants of health as a link between health and social resources, which can include substance use disorder services.

## Data Availability

Not applicable.

## Abbreviations

CHAG: Community Health Awareness Group Inc. Detroit
CMH: Community Mental Health
DHS: Department of Human Services
LGBTQIA+: Lesbian, Gay, Bisexual, Transgender, Queer, Intersex, Asexual+
MDHSS: Michigan Department of Health and Human Services
NESARC-III: National Epidemiologic Survey of Alcohol and Related Conditions–III
NSDUH: National Survey on Drug Use and Health
PHRL: Public Health Research Lab
SPSS 27: Statistical Package for the Social Sciences 27
SSP: Syringe Exchange Programs
STR: State Targeted Response
SUD: Substance Use & Substance Use Disorders
US: United States
WSU: Wayne State University

## References

[1] Spencer MR, Minino AM, Warner M. Drug Overdose Deaths in the United States, 2001–2021: Centers for Disease Control and Prevention (CDC); 2022 [Available from: https://www.cdc.gov/nchs/products/databriefs/db457.htm.

[2] Pasareanu AR, Opsal A, Vederhus JK, Kristensen O, Clausen T (2015). Quality of life improved following in-patient substance use disorder treatment. Health Qual Life Outcomes.

[3] Pinedo M, Villatoro AP (2020). The role of perceived treatment need in explaining racial/ethnic disparities in the use of substance abuse treatment services. J Subst Abuse Treat.

[4] Urada D, Teruya C, Gelberg L, Rawson R (2014). Integration of substance use disorder services with primary care: health center surveys and qualitative interviews. Subst Abuse Treat Prev Policy.

[5] Pullen E, Oser C (2014). Barriers to substance abuse treatment in rural and urban communities: counselor perspectives. Subst Use Misuse.

[6] Brook RD, Dawood K, Foster B, Foust RM, Gaughan C, Kurian P (2022). Utilizing Mobile Health Units for Mass Hypertension Screening in socially vulnerable communities across Detroit. Hypertension.

[7] Murphy DC, Klinghoffer I, Fernandez-Wilson JB, Rosenberg L (2000). Mobile health units. Design and implementation considerations. AAOHN J.

[8] Muncan B, Walters SM, Ezell J, Ompad DC (2020). They look at us like junkies: influences of drug use stigma on the healthcare engagement of people who inject drugs in New York City. Harm Reduct J.

[9] Paquette CE, Syvertsen JL, Pollini RA (2018). Stigma at every turn: health services experiences among people who inject drugs. Int J Drug Policy.

[10] Krawczyk N, Allen ST, Schneider KE, Solomon K, Shah H, Morris M (2022). Intersecting substance use treatment and harm reduction services: exploring the characteristics and service needs of a community-based sample of people who use drugs. Harm Reduct J.

[11] Claborn K, Samora J, McCormick K, Whittfield Q, Courtois F, Lozada K (2023). We do it ourselves: strengths and opportunities for improving the practice of harm reduction. Harm Reduct J.

[12] Khan GK, Harvey L, Johnson S, Long P, Kimmel S, Pierre C (2022). Integration of a community-based harm reduction program into a safety net hospital: a qualitative study. Harm Reduct J.

[13] Nakaishi L, Sugden SG, Merlo G (2023). Primary care at the intersection of Lifestyle interventions and Unhealthy Substance Use. Am J Lifestyle Med.

[14] Oriol NE, Cote PJ, Vavasis AP, Bennet J, Delorenzo D, Blanc P (2009). Calculating the return on investment of mobile healthcare. BMC Med.

[15] Levy P, McGlynn E, Hill AB, Zhang L, Korzeniewski SJ, Foster B (2021). From pandemic response to portable population health: a formative evaluation of the Detroit mobile health unit program. PLoS ONE.

[16] Bowles KE, Clark HA, Tai E, Sullivan PS, Song B, Tsang J (2008). Implementing rapid HIV testing in outreach and community settings: results from an advancing HIV prevention demonstration project conducted in seven U.S. cities. Public Health Rep.

[17] Clark HA, Bowles KE, Song B, Heffelfinger JD (2008). Implementation of rapid HIV testing programs in community and outreach settings: perspectives from staff at eight community-based organizations in seven U.S. cities. Public Health Rep.

[18] Gleason Comstock J. Development of a CBPR Protocol for an IDU Focus Group on Overdose Prevention and Naloxone Training & Distribution. Mich J Public Health. 2010;4(1).

[19] Gleason Comstock J, M G, Lewis SY. C. Substance Use Disorder Services (SUDS) Evaluation Community Health Awareness Group. 2021.

[20] Streater A, Gleason Comstock J. Life Points Harm Reduction Outreach Project FY2011-2012. Evaluation Report. 2012.

[21] Gleason Comstock J, Streater A, Bolden CC. L B. Community-Based Participatory Research with Intravenous Drug Users on Overdose Reversal [Research Product]. CES4Health.info! 2010 [2010:[Available from: http://www.ces4health.info/find-products/view-product.aspx?code=4RD8X6YX.

[22] Gleason-Comstock J, Streater A, Bolden Calhoun C, Norman SJ. A syringe exchange program as a public health point of contact. Wayne State University President’s Biennial Conference on health Disparities; Detroit2007.

[23] McCabe SE, Bostwick WB, Hughes TL, West BT, Boyd CJ (2010). The relationship between discrimination and substance use disorders among lesbian, gay, and bisexual adults in the United States. Am J Public Health.

[24] Social Determinants of Health at CDC: Centers for Disease Control and Prevention. 2022 [Available from: https://www.cdc.gov/about/sdoh/index.html.

[25] Feinstein EC, Richter L, Foster SE (2012). Addressing the critical health problem of adolescent substance use through health care, research, and public policy. J Adolesc Health.

